# Quantifying the efficiency of Hydroxyapatite Mineralising Peptides

**DOI:** 10.1038/s41598-017-07247-z

**Published:** 2017-08-09

**Authors:** Robyn Plowright, David J. Belton, David L. Kaplan, Carole C. Perry

**Affiliations:** 1 Biomolecular and Materials Interface Research Group, Interdisciplinary Biomedical Research Centre, School of Science and Technology, Nottingham Trent University, Clifton Lane, Nottingham, NG11 8NS UK; 2 Department of Biomedical Engineering, Tufts University, 4 Colby Street, Medford, Massachusetts 02155 United States

## Abstract

We present a non-destructive analytical calibration tool to allow quantitative assessment of individual calcium phosphates such as hydroxyapatite (HAP) from mixtures including brushite. Many experimental approaches are used to evaluate the mineralising capabilities of biomolecules including peptides. However, it is difficult to quantitatively compare the efficacy of peptides in the promotion of mineralisation when inseparable mixtures of different minerals are produced. To address this challenge, a series of hydroxyapatite and brushite mixtures were produced as a percent/weight (0–100%) from pure components and multiple (N = 10) XRD patterns were collected for each mixture. A linear relationship between the ratio of selected peak heights and the molar ratio was found. Using this method, the mineralising capabilities of three known hydroxyapatite binding peptides, CaP(S) STLPIPHEFSRE, CaP(V) VTKHLNQISQSY and CaP(H) SVSVGMKPSPRP, was compared. All three directed mineralisation towards hydroxyapatite in a peptide concentration dependent manner. CaP(V) was most effective at inducing hydroxyapatite formation at higher reagent levels (Ca^2+^ = 200 mM), as also seen with peptide-silk chimeric materials, whereas CaP(S) was most effective when lower concentrations of calcium (20 mM) and phosphate were used. The approach can be extended to investigate HAP mineralisation in the presence of any number of mineralisation promoters or inhibitors.

## Introduction

The mineralising potential of specific binding peptides has been studied for a wide range of materials and reaction conditions^1, 2^. Methods that allow accurate quantification of the mineralised products tend to have one of two distinguishing features; either only one product is formed (or not formed) or the by-products of the experiment are easily separated from the target compound.

In peptide driven hydroxyapatite (HAP) synthesis it is often the case that inseparable mixtures of various calcium phosphate crystalline materials are produced^3^. The range of crystal phases produced varies with the method of mineralisation employed, however the more commonly used methods tend to be the simplest, and result in two significant phases forming, namely HAP and brushite. Analysis of the efficiency of peptide directed mineralisation that generates impure products/mixtures is then limited to a qualitative discussion of products, for example ‘products produced in the presence of sample 1 appears to contain a greater fraction of HAP than sample 2′.

In this study a series of HAP/brushite mixtures with increasing molar ratios of HAP to brushite (the most commonly formed calcium phosphate without mineralising directing influences present)^4–7^ have been analysed with the intention of creating an analytical method applicable to the quantifiable estimation of, in this case, peptides that promote HAP mineralisation. Three HAP binding peptides, all of which were previously identified *via* phage display: CaP(S) STLPIPHEFSRE^8–11^, CaP(V) VTKHLNQISQSY^10, 12–15^ and CaP(H) SVSVGMKPSPRP^8, 13, 16, 17^, have been used in this study. An extension of the approach to quantify the effect on mineralisation of larger proteins such as silk chimeras built from mimics of spider silk MASP1 protein and the CaP(V) peptide is also included^18^.

## Method

### Brushite Synthesis

Equal volumes of saturated aqueous solutions of 200 mM calcium chloride (Sigma-Aldrich, Dorset UK) and 120 mM dibasic sodium phosphate (Sigma-Aldrich, Dorset UK) were added together and the solution stirred for 1 hour. The precipitate was collected and washed *via* centrifugation and sonication using dd water. A total of 5 washes were performed. Precipitates were dried by lyophilisation (24 hours).

### Producing Mixtures

Hydroxyapatite (Sigma-Aldrich, Dorset, UK) and brushite (synthesised for this study) were mixed according to the percentage weight of each: 0, 20, 40, 50, 60, 70, 80, 90 and 100%. The full range was produced to allow use in the study of mineralisation with increasing concentrations of known HAP binding peptides and non-directed (control) mineralisation. A total of 10 XRD patterns were collected for each mixture with thorough stirring between each scan.

### Assessment of crystalline phases present by powder x-ray diffraction

XRD (PANalytical X’Pert PRO, Cu Kα radiation with wavelength of 1.54056 Å) was used to characterise the crystallinity of the brushite precipitates produced and mixtures formed from brushite and HAP. Aluminium sample holders were packed with polydimethylsiloxane (PDMS) (Sylgard 184 PDMS, Mlsolar, Campbell, CA, USA), and samples scanned from 5° to 65° of 2θ, accelerating voltage 45 kV, filament current 40 mA and scanning speed 0.02° s^−1^. X’PertHighScore Plus (Version 2.0a) was used for pattern manipulation (baseline correcting and smoothing) and analysis. Artificially generated mixtures were analysed 10x and materials generated in the presence of peptides were analysed 3 times with mixing of the precipitate between each scan with samples being kept in the lyophilising chamber until analysis.

### Infra-red Spectroscopy

FTIR-ATR (Frontier, PerkinElmer, Coventry, UK), was performed to identify the presence of phosphate groups in initial materials, mixtures and mineralised products generated in the presence of peptides. For each sample, an average of 40 scans were collected over the range 4000–650 cm^−1^ at 2 cm^−1^ resolution.

### Inductively Coupled Plasma – Optical Emission Spectroscopy

The molar ratio of HAP to Brushite was assessed *via* the Ca:P ratio determined from ICP, with data normalised with respect to 100% HAP and 100% Brushite. Approximately 5 mg of each HAP/Brushite standard (the same standards as used in XRD analysis), was digested in 1 mL aqua regia overnight then diluted 10 fold using dd. water. Measurements are stated as an average of 3 readings per sample.

### Hydroxyapatite synthesis using binding peptides

1 mL aqueous solutions of each peptide; CaP(S) STLPIPHEFSRE, CaP(V) VTKHLNQISQSY and CaP(H) SVSVGMKPSPRP (Pepceuticals Limited, Leicester, UK −>95% purity), were produced at concentrations of: 0.1 mg/mL, 0.25 mg/mL, 0.5 mg/mL, 1 mg/mL, 2 mg/mL and 4 mg/mL. Each solution had equal volumes (4 mL) of calcium chloride 200 mM (Sigma-Aldrich, Dorset, UK) and dibasic sodium phosphate 120 mM (Sigma-Aldrich, Dorset, UK) added in 200 μL aliquots and solutions were kept at circumneutral pH, SI Figure 2. After addition of all reagents, solutions were left to stir for 1 hour before washing the precipitate 3 times with dd water. Synthesis of mineralised products was then repeated using lower reagent concentrations, namely 20 mM calcium chloride and 12 mM dibasic sodium phosphate^19–23^.

## Results

XRD was selected for this study as it is a well-established non-destructive technique for the identification of HAP. Patterns produced from highly crystalline materials require little processing so there is a minimal risk of loss of information^24^. IR and Raman spectroscopy were excluded as both require a higher level of processing (baseline correction followed by peak deconvolution of phosphate peaks) on the spectra to gain quantifiable data. The peaks present in the IR spectra of HAP and Brushite are phosphate groups and cannot be easily classified as arising from HAP or Brushite. ICP, as a destructive technique, can measure total ion concentrations but cannot quantify the ions arising from a specific phase, though the possibility of normalising measured Ca:P ratios from ICP data is explored here, assuming 100% HAP is 1.67 and 100% Brushite is 1.

### Confirmation of Brushite synthesis

X-ray diffraction (XRD) and infra-red spectroscopy (IR) were used to confirm the formation of Brushite. The XRD pattern showed the distinctive Brushite pattern whilst IR confirmed the presence of phosphate groups with minimal water content in the sample, (SI Figure 1).

### Development of a method to assess Hydroxyapatite/Brushite mixtures

Powder x-ray diffraction has been extensively used for HAP identification since Dejong first observed the difference between bone powder and standard highly crystalline HAP^25^. The crystal structure of HAP is an hexagonal system, the space group is P6_3_/m with lattice parameters a = b = 9432 Å and c = 6.884 Å with γ = 120°. This gives a complex and distinct XRD pattern that allows in depth analysis where defects in structure can be determined^26, 27^. For quantification of the relative amounts of each phase present in mixed samples, a single intense peak was selected from each pattern; the (020) peak at approximately 12° for Brushite and initially the (211) peak for HAP at approximately 32° to allow smaller quantities of either material to be detected more easily. Once a trend was established the (002) peak at 25° two theta was then used for HAP as there is no risk of peak merging, a common occurrence in naturally produced (non-heat treated) HAP XRD patterns due to the smaller crystal sizes present and/or the non-stoichiometric nature of the sample^28^.

**Figure 1:**
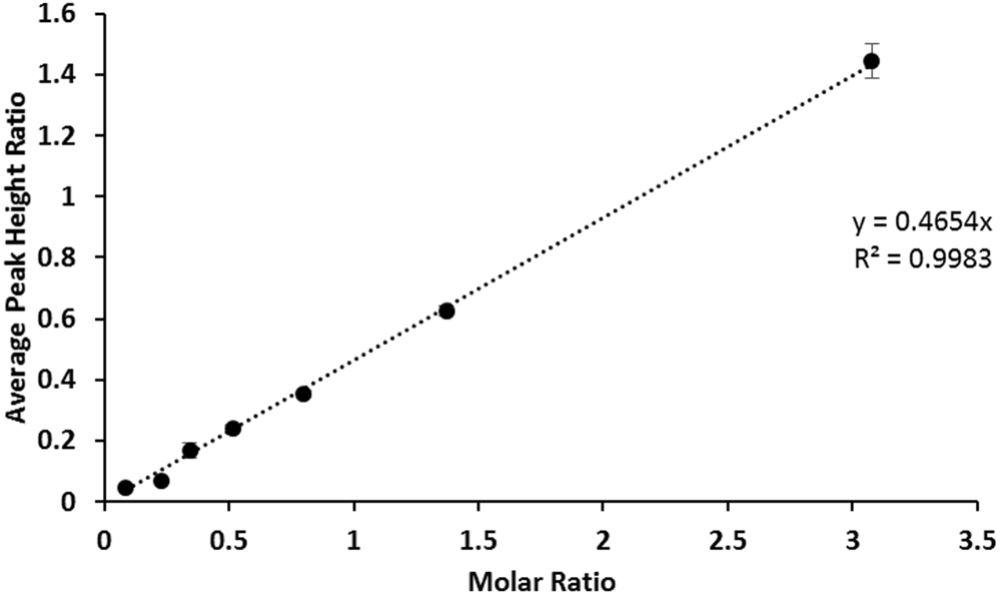
Average peak height ratio for the (020) Brushite peak vs the (002) HAP peak versus the molar ratio of HAP: Brushite, n = 10. R^2^ = 0.9983.

Mixtures of HAP and Brushite at pre-defined ratios were produced and studied by XRD to establish whether a relationship exists between information extractable from the XRD patterns and the molar ratio of the two calcium phosphates present in an individual sample. Relationships between concentration and peak height or peak area for the two phases were compared Fig. 1 and SI Figure 2. Analysis using peak height ratios gave the clearer relationship with a clear linear trend between the average peak height ratio for the (020) Brushite peak vs the (002) HAP peak and the molar ratio of HAP to Brushite for each sample being observed, Fig. 1. The trend can be visualised in example XRD patterns for each mixture (SI Figure 3) which show that (i) the Brushite (020) peak at 12° decreases in intensity with increasing HAP concentration, and (ii) the most intense HAP peak at 32° increases in intensity with increasing HAP concentration. Additional confidence in the approach arises from the very small variations in the values obtained from multiple independent (N = 10) sample measurements.

### Quantitative comparison of the ability of hydroxyapatite binding peptides to affect mineralisation

Three known HAP binding peptides, previously identified by phage display were included in the study to see if the mineralising capabilities of each could be measured quantitatively. The effect of peptide concentration and reagent concentrations were both explored. The three peptides, (CaP(S) STLPIPHEFSRE, CaP(V) VTKHLNQISQSY and CaP(H) SVSVGMKPSPRP)^8, 16^ had all previously been identified as HAP binding peptides. Initial experiments were carried out using the same concentrations of calcium chloride (200 mM) and dibasic sodium phosphate (120 mM) as used to generate brushite for the development of the approach.

**Figure 2:**
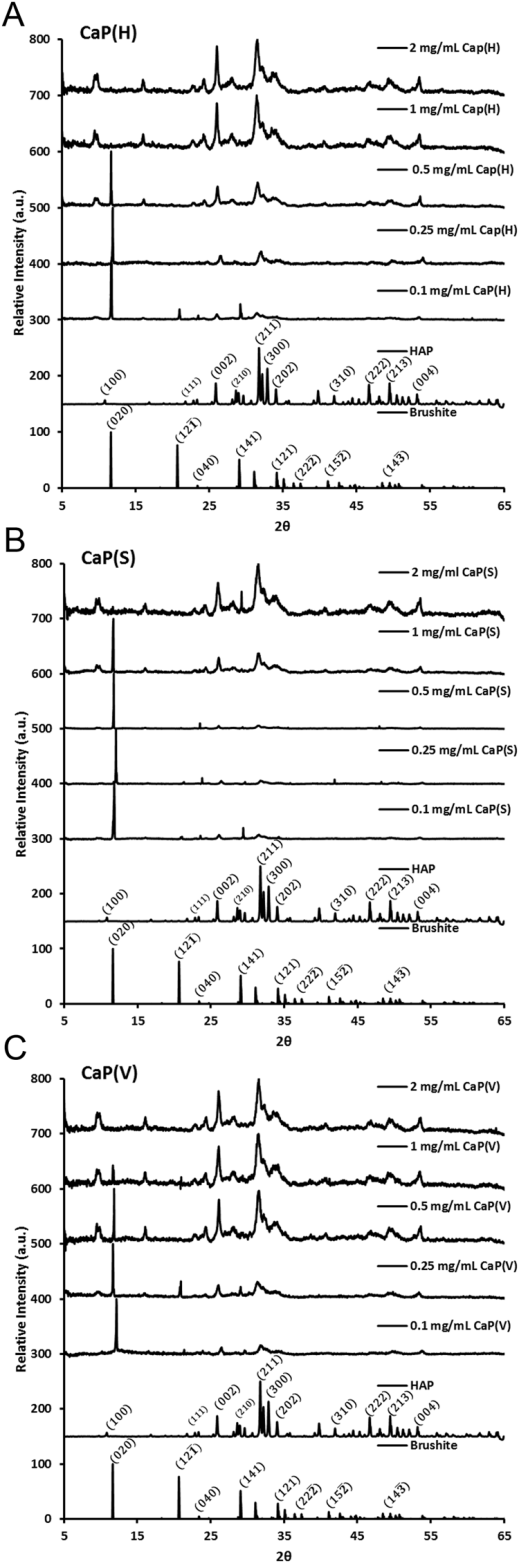
(**A**–**C**) XRD patterns of precipitates formed from reaction of 200 mM calcium chloride and 120 mM dibasic sodium phosphate in the presence of CaP(H) SVSVGMKPSPRP, CaP(S) STLPIPHEFSRE and CaP(V) VTKHLNQISQSY at initial concentrations: 0.1, 0.25, 0.5, 1 and 2 mg/mL.

All three peptides were able to direct mineralisation towards HAP formation. Figure 2A–C shows the XRD patterns produced from precipitates formed in the presence of each peptide at initial concentrations; 0.1 mg/mL, 0.25 mg/mL, 0.5 mg/mL, 1 mg/mL and 2 mg/mL. Table 1 shows the estimated percentages of HAP present in each precipitate formed. There is a clear increase in the percentage of HAP produced with increasing concentration of all peptides. In the XRD patterns this can be visualised by the decrease in intensity of the (020) Brushite peak and an increase in the (002) and (211) peaks of HAP.

**Table 1:** Calculated percentage mass of HAP in each precipitate.

Concentration of peptide used	Estimated Percentage HAP Produced		
	CaP(H)	CaP(S)	CaP(V)
0.1 mg/mL	39.11 ± 1.13	33.11 ± 0.96	43.40 ± 1.26
0.25 mg/mL	47.14 ± 1.36	27.21 ± 0.79	58.84 ± 1.70
0.5 mg/mL	67.19 ± 1.95	26.39 ± 0.77	82.80 ± 2.40
1 mg/mL	100 ± 0	62.09 ± 1.80	99.30 ± 0.54
2 mg/mL	100 ± 0	100 ± 0	100 ± 0

.

As the percentage of HAP increases with initial peptide concentration it suggests that the concentration of peptide used is a key parameter in HAP mineralisation. This will reach a limit, however, when all precipitated material is present as HAP as seen in this study for CaP(V) and CaP(H) when added to the initial reaction at a level above 1 mg/ml and for additions of CaP(s) at 2 mg/ml. From this data it suggests that for all such peptides a maximum limit on the amount of peptide required for HAP mineralisation can be ascertained using XRD as a quantitative tool. The efficacy of the peptides in promoting mineralisation can be readily compared, with data on CaP(S) and CaP(V) supporting earlier literature studies where more CaP(V) was found to bind to HA than CaP(S)^12, 13, 29^.

Addition of either CaP(V) and Cap(H) led to a greater degree of control over mineralisation at the lower concentrations used, (at 1 mg/mL 99% and 100% HAP was produced respectively compared with CaP(S) at 62%). CaP(V) was shown to have more influence at the lowest concentrations explored at 0.1 mg/mL and 0.25 mg/mL. An argument could be made that CaP(V) has greater influence and is therefore better at inducing HAP formation, however, all of these peptides could be used providing an appropriate concentration level is selected for each, information which may be useful for peptide selection if charge or hydrophobicity are important factors.

### Quantitative comparison of hydroxyapatite binding peptide with limited reagents

**Figure 3:**
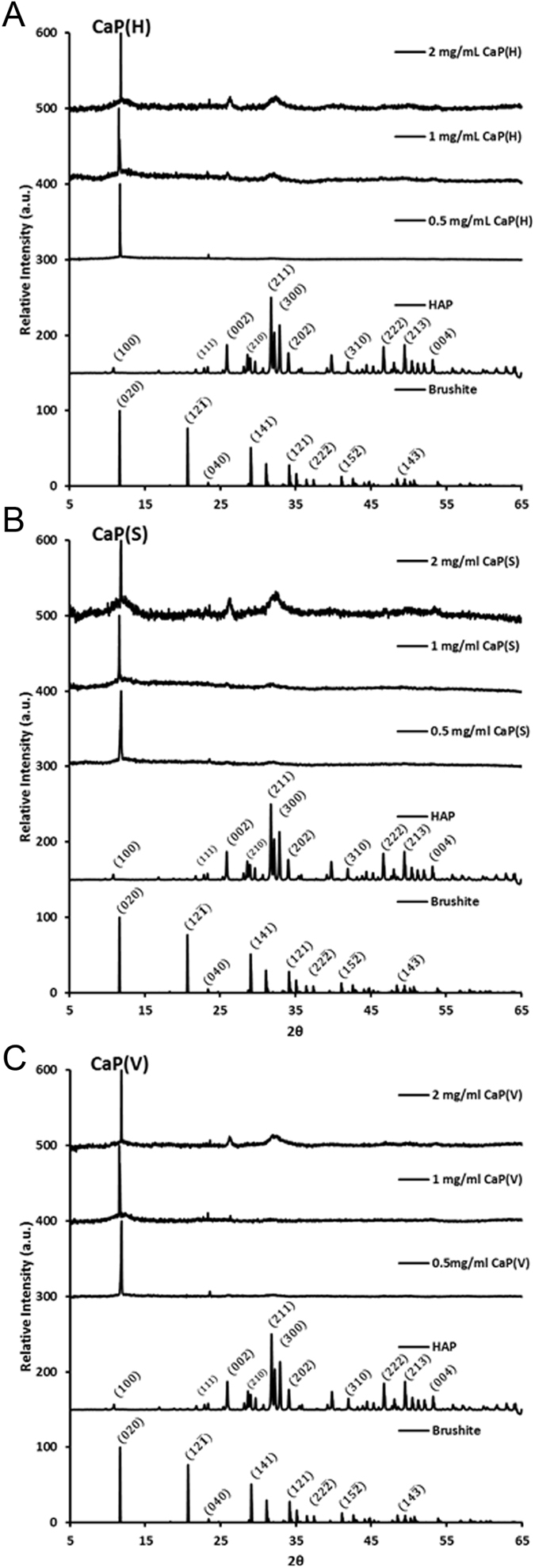
(**A**–**C**) XRD patterns of the precipitates formed from the reaction of 20 mM calcium chloride and 12 mM sodium phosphate dibasic in the presence of CaP(S) STLPIPHEFSRE, CaP(V) VTKHLNQISQSY and CaP(H) SVSVGMKPSPRP at initial concentrations: 0.5, 1 and 2 mg/mL.

Once each peptide was shown to be capable of inducing HAP formation, the concentration of each reagent was reduced tenfold and the experiments repeated as above to determine if the three selected HAP binding peptides could still influence mineralisation under much reduced ion concentrations. The peptide concentrations were chosen to replicate effects (differences in mineralisation activity for the various peptides) observed at the lower end of the of peptide concentration range used in the initial experiments. Figure 3A–C shows the XRD patterns for precipitates produced in the presence of the peptides; CaP(H), CaP(S) and CaP(V) at a range of initial concentrations (0.5–2 mg/ml) and Table 2 shows the estimated percentages of HAP present in the precipitates. All of the peptides influenced precipitation particularly as the initial concentration of peptide was increased. Under these conditions, the maximum level of HAP formation in the precipitates was of the order of 50%. In contrast to results obtained for the effect of peptide on HAP formation at ‘high’ reagent concentrations, Fig. 2, here CaP(S) was the most effective HAP ‘promoter’ at the lowest reagent concentration level used. The reasons for the different behaviour of CaP(H), CaP(S) and CaP(V) probably relate to the specific properties of the peptides themselves and will be discussed later in this article.

**Table 2:** Calculated percentage mass of HAP in each precipitate (it was not possible to obtain data from multiple samples owing to the very small amount of sample generated).

Concentration of peptide used	Estimated Percentage HAP Produced		
	CaP(H)	CaP(S)	CaP(V)
0.5 mg/mL	5	3	6
1 mg/mL	41	41	21
2 mg/mL	49	57	44

### Alternative Techniques

#### Infrared Spectroscopy

**Figure 4:**
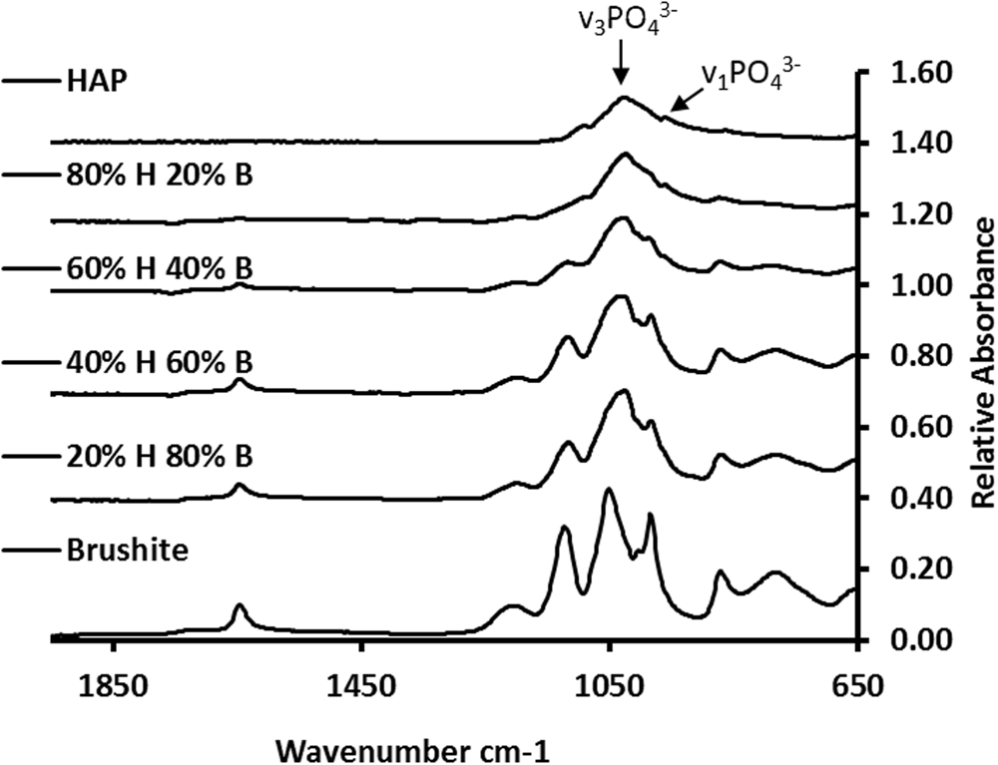
Example FTIR (ATR) spectra for a selection of the HAP/Brushite mixtures.

FTIR spectra was used to confirm the presence of phosphates for the mixtures and precipitates formed in the presence of a mineralising peptides, Fig. 4, phosphate peaks: 950 cm^−1^ *v*_1_(PO_4_^3−^), 1010 cm^−1^ *v*_3_(PO_4_^3−^). However, this data cannot be readily used quantitatively, due to the effect of water on the spectra and (i) the difficulty of measuring peak heights from a baseline that fluctuates significantly more than that of XRD, or (ii) problems due to significant overlap between peaks making deconvolution difficult.

#### Inductively couple plasma-Optical Emission Spectrometry

**Figure 5:**
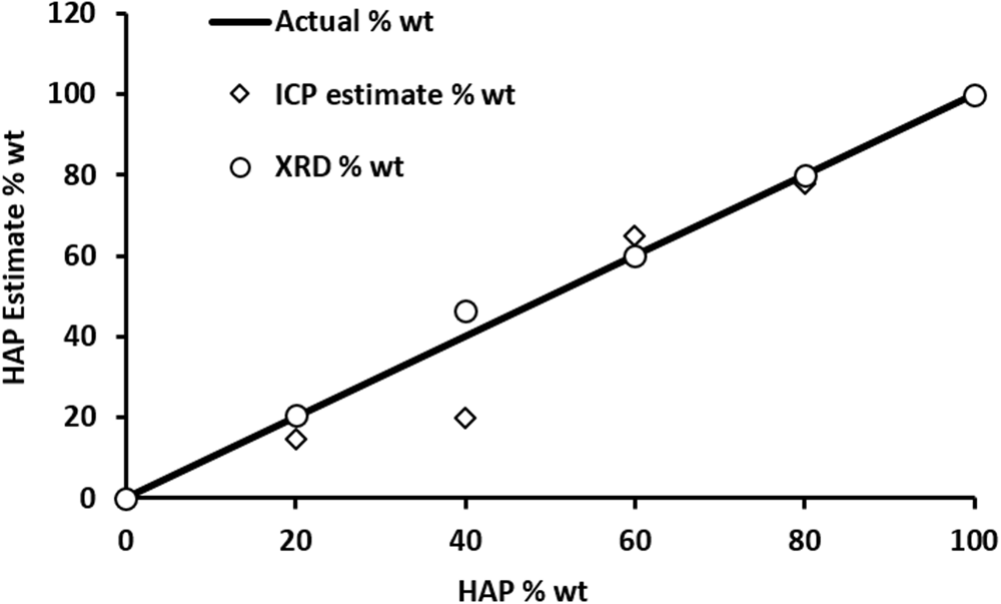
Exact percentage of HAP present in standards plotted against the measured percentage from the different analytical techniques employed: ICP and XRD.

Measurement of the same standards used in this study were also carried out *via* ICP-OES to find the Ca:P ratio of samples. Once normalised to the 0 and 100% HAP sample values, there was a clear increase in the molar ratio with increased HAP percentage, Fig. 5, and Supplementary Table 1. However this method proved unreliable with phase composition being both over and underestimated. It is believed ICP is not an appropriate technique for phase identification based on the data and the assumptions that must be made with ICP: only the two crystal phases HAP and Brushite are present and that the calcium to phosphorus ratio is based on defect free structures.

### Extension of approach to silk chimeras

Previous published work has focused on one of the mineralising peptides, CaP(V), as part of a genetically engineered silk material^18^. The prior study showed that increased number of peptides attached to the silk backbone resulted in greater control over mineralisation. However, discussion of the diffraction data was limited to statements of ‘increase or reduction’ in HAP and brushite peaks respectively. Using the analytical method described in this contribution, Table 3 we can show that silk constructs based on spider silk MASP1 with a His tag are able to promote HAP formation. The location of the peptide at either the N or C terminus had little effect on the extent of mineralisation but the presence of peptide at both ends of the fusion protein leads to enhanced mineralisation. What is particularly impressive is the low concentration of peptide, when attached to the silk construct required to generate pure HAP samples, at 0.033 mg/ml as compared to when the peptide is used alone when a 1 mg/ml solution is required.

**Table 3:** Previously published data on silk-fusion proteins containing the CaP(V) (or VTK) binding peptide with new quantification method applied^18^. nh refers to His tag at N-terminus of protein; ch refers to His tag at C-terminus of protein; 15mer refers to 15 repeats of the spider silk modified MASP1 33 amino acid sequence.

Sample 1 mg/mL	%/wt HAP	Concentration VTK-15mer-VTK mg/mL	%/wt HAP
nh-15mer	32	0.25	59
nh-15mer-VTK	63	0.5	87
15mer-ch	44	0.75	92
VTK-15mer-ch	62	1	100
VTK-15mer-VTK	100	2	100
		3	100

### Mechanism of peptide directed mineralisation

**Figure 6:**
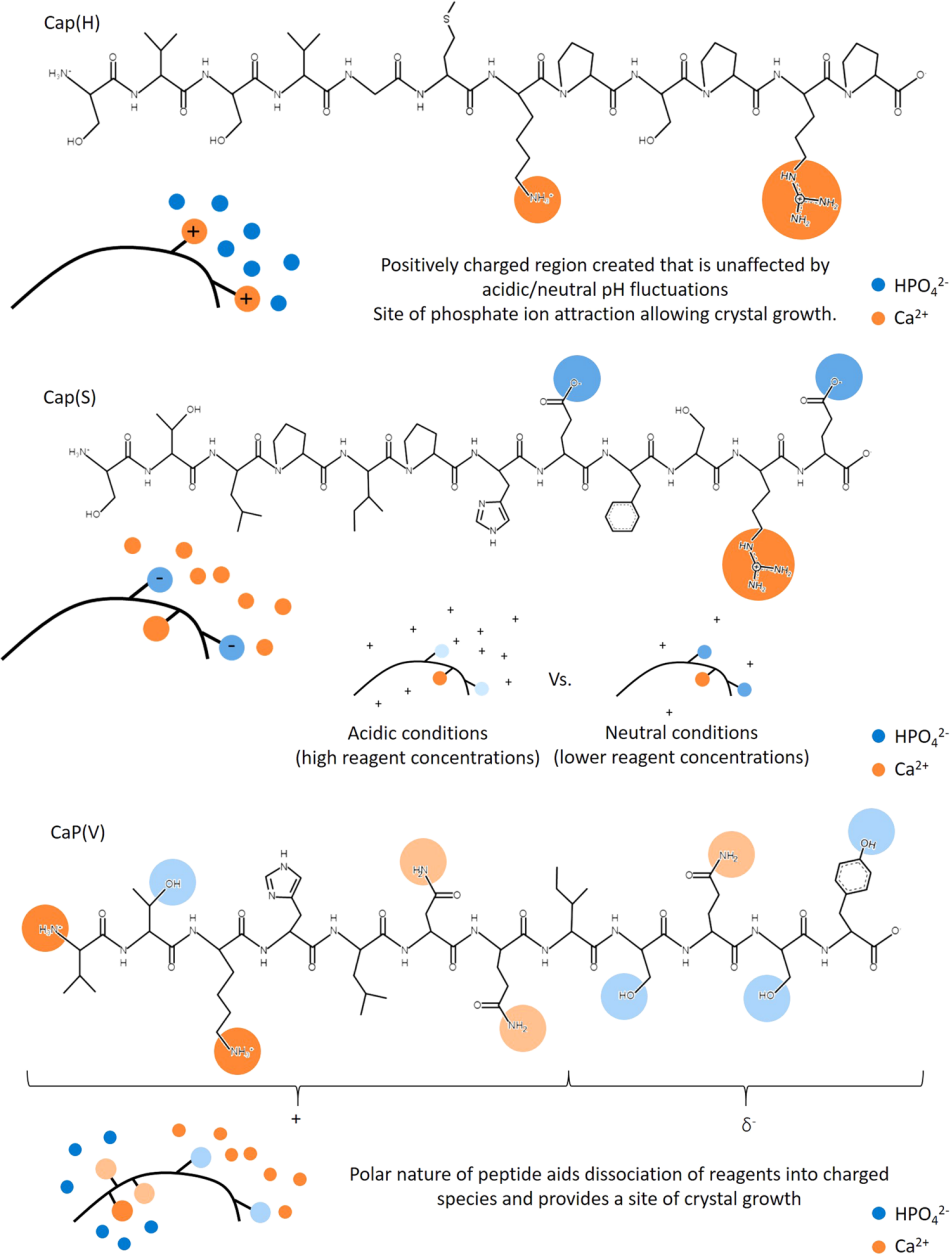
Influences of each peptide over mineralisation with respect to charged species and pH.

Now we consider the role of the individual peptides and their response during mineralisation, Fig. 6. Note, prior research has shown that scrambling of the sequence of CaP(V) had little effect on peptide binding to a range of synthetic bone-like and apatitic materials^12^. At higher reagent concentrations (200 mM CaCl_2_ and 120 mM NaHPO_4_), additions of CaP(H) and CaP(V) showed greater control over mineralisation, and as both peptides have a net positive charge it is suggested that their addition drives mineralisation through phosphate ion attraction. Under the conditions of the mineralisation experiments, despite CaP(H) having a + 2 charge and CaP(V) only +1 charge, SI Figure 4, it is likely that CaP(V) is able to rival the mineralising capabilities of CaP(H) as the positively charged lysine group resides at the amino terminal of the peptide, thereby creating an area of high charge density; this coupled with the large number of hydroxyl groups at the carboxyl terminal, gives the peptide a polar quality. CaP(V) would be able to attract both cations and anions thereby providing a site for crystal growth.

CaP(S) had less of an effect over mineralisation compared to CaP(H) and CaP(V) at high ion concentrations, which we suggest is due to the overall negative charge of CaP(S) and small changes in pH away from neutral during the mineralisation reaction preventing organisation of cations and anions in proximity to the peptide or protein. At lower ion concentrations, this effect is felt less and mineralisation where cation organisation is key most likely dominates, with CaP(S) becoming a good mineralisation promoter. It is possible that the differing physical properties of CaP(S) (negative overall charge with an acidic calculated isoelectric point) compared with CaP(H) and CaP(V) may allow for a more stable unfolded protein structure in solution which would allow for greater exposure of amine groups and therefore greater promotion of mineralisation.

## Conclusions

We have developed an analytical calibration tool to quantitatively estimate the % HAP in mixed precipitates and shown that the method can be applied to compare the mineralisation efficacy of peptides and fusion proteins. The general approach should be applicable to other mineralisation systems where mixed phases are commonly produced and assessment of the role(s) of additives in mineralisation control ﻿is desired. The production of mixtures of HAP and Brushite (known concentrations) allowed a direct correlation between average peak height (XRD) and molar ratio to be found. Using this calibration method, it was possible to compare the activity of three known HAP binding peptides. Although it was found CaP(V) showed better control over mineralisation at lower concentrations of peptide, it is believed all peptides included in the study would be suitable choices as HAP binders providing an appropriate concentration is used, meaning other aspects such as physical properties of the biomolecules themselves could also be considered.

## Electronic supplementary material

Supplementary Information 

## References

[1] Sigel A, Sigel H, Sigel R (2010). Biomineralisation: From Nature to Application.

[2] Mann S (2001). Biomineralization: Principles and Concepts in Bioinorganic Materials Chemistry.

[3] Furedimi H, Purgaric B, Purgaric B, Pavkovic N (1971). Precipitation of Calcium Phosphates from Electrolyte Solutions. Study of Precipitates in Physiological pH Region. Calc Tiss Res.

[4] Eanes ED, Gillessen IH, Posner AS (1965). Intermediate states in the precipitation of hydroxyapatite. Nature.

[5] Walsh G (2002). Proteins Biochemistry and Biotechnology.

[6] Shiryaev M, Safronova T, Putlyaev V (2010). Calcium phosphate powders synthesized from calcium chloride and potassium hydrophosphate. J Therm Anal Calorim.

[7] Safronova TV, Kuznetsov AV, Korneychuk SA, Putlyaev VI, Shekhirev MA (2009). Calcium phosphate powders synthesized from solutions with [Ca^2+^]/[PO_4_^3−^] = 1 for bioresorbable ceramics. Cent Eur J Chem.

[8] Segvich S, Biswas S, Becker U, Kohn DH (2009). Identification of Peptides with Targeted Adhesion to Bone-Like Mineral via Phage Display and Computational Modeling. Cells Tissues Organs (Print).

[9] Chung, C., Park, Y., Rhee, S. H. & Lee, J. Y. Peptide having the ability to regenerate bone tissue and for binding to apatite (2013).

[10] Segvich, S., J. Design of Peptides with Targeted Apatite and Human Bone Marrow Stromal Cell Adhesion for Bone Tissue Engineering (2009).

[11] Zhang S (2011). Biological and biomedical coatings handbook: processing and characterization.

[12] Addison WN (2010). Phosphorylation-dependent mineral-type specificity for apatite-binding peptide sequences. Biomaterials.

[13] Puleo DA, Bizios R (2009). Biological interactions on materials surfaces: understanding and controlling protein, cell, and tissue responses.

[14] Ramaraju H, Miller SJ, Kohn DH (2014). Dual-functioning phage-derived peptides encourage human bone marrow cell-specific attachment to mineralized biomaterials. Connect. Tissue Res..

[15] Subramanian, G. In *Biopharmaceutical production technology* (Wiley, 2012).

[16] Roy MD, Stanley SK, Amis EJ, Becker ML (2008). Identification of a highly specific hydroxyapatite-binding peptide using phage display. Adv Mater.

[17] Bronner F, Farach-Carson MC, Roach HI (2010). Bone and development.

[18] Dinjaski N (2017). Osteoinductive recombinant silk fusion proteins for bone regeneration. Acta Biomater.

[19] Jahromi MT, Yao G, Cerruti M (2013). The importance of amino acid interactions in the crystallization of hydroxyapatite. J Roy Soc Iinterface.

[20] Jahromi MT, Cerruti M (2015). Amino Acid/Ion Aggregate Formation and Their Role in Hydroxyapatite Precipitation. Cryst Growth Des.

[21] Pan H, Tao J, Xu X, Tang R (2007). Adsorption processes of Gly and Glu amino acids on hydroxyapatite surfaces at the atomic level. Langmuir.

[22] Spanos N, Klepetsanis P, Koutsoukos P (2001). Model studies on the interaction of amino acids with biominerals: The effect of L-serine at the hydroxyapatite-water interface. J. Colloid Interface Sci..

[23] Tao J, Pan H, Zeng Y, Xu X, Tang R (2007). Roles of amorphous calcium phosphate and biological additives in the assembly of hydroxyapatite nanoparticles. J Phys Chem B.

[24] Selvig KA (1972). Crystal-Structure of Hydroxyapatite in Dental Enamel as seen with Electron-Microscope. J. Ultrastruct. Res..

[25] de Jong WF (1926). La Substance Minérale Dans les Os. RECL Recueil des Travaux Chimiques des Pays-Bas.

[26] Chaudhry AA (2008). Synthesis and characterisation of magnesium substituted calcium phosphate bioceramic nanoparticles made via continuous hydrothermal flow synthesis. J Mater Chem.

[27] Panda R, Hsieh M, Chung R, Chin T (2003). FTIR, XRD, SEM and solid state NMR investigations of carbonate-containing hydroxyapatite nano-particles synthesized by hydroxide-gel technique. J Phys Chem Solids.

[28] Mobasherpour I, Heshajin MS, Kazemzadeh A, Zakeri M (2007). Synthesis of nanocrystalline hydroxyapatite by using precipitation method. J Alloy Compd.

[29] Segvich SJ, Smith HC, Kohn DH (2009). The adsorption of preferential binding peptides to apatite-based materials. Biomaterials.

