# Supplementary material for: Quantifying the efficiency of Hydroxyapatite Mineralising Peptides
Source: Sci Rep. 2017 Aug 9;7:7681. doi: 10.1038/s41598-017-07247-z (PMC5550443; doi:10.1038/s41598-017-07247-z)
Supplement: Supplementary file 1 — Supplementary Information [file 41598_2017_7247_MOESM1_ESM.pdf]

Quantifying the efficiency of Hydroxyapatite Mineralising Peptides

Robyn Plowright[1], David J. Belton[1], David L. Kaplan[2] and Carole C. Perry[1]*

[1]Biomolecular and Materials Interface Research Group, Interdisciplinary Biomedical Research Centre, School of Science and Technology, Nottingham Trent University, Clifton Lane, Nottingham, UK NG11 8NS.

[2]Department of Biomedical Engineering, Tufts University, 4 Colby Street, Medford, Massachusetts, 02155, United States.

* To whom correspondence should be addressed:

Carole C. Perry. E-mail carole.perry@ntu.ac.uk , Tel. +44 115 84 86695

Supplementary Information

Confirmation of Brushite formation

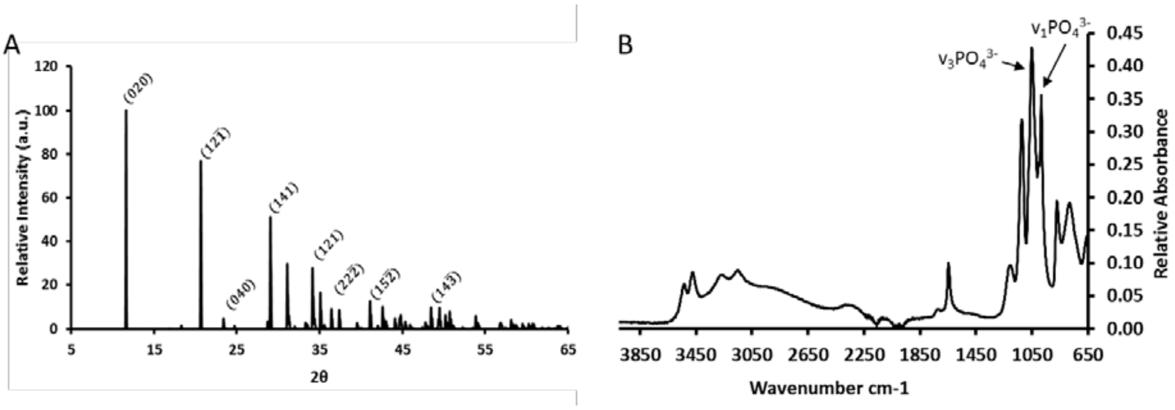

SI Figure 1 Confirmation of the presence of brushite A) XRD B) FTIR (ATR).

Average Peak Area Ratio vs. Molar Ratio of CaP phases

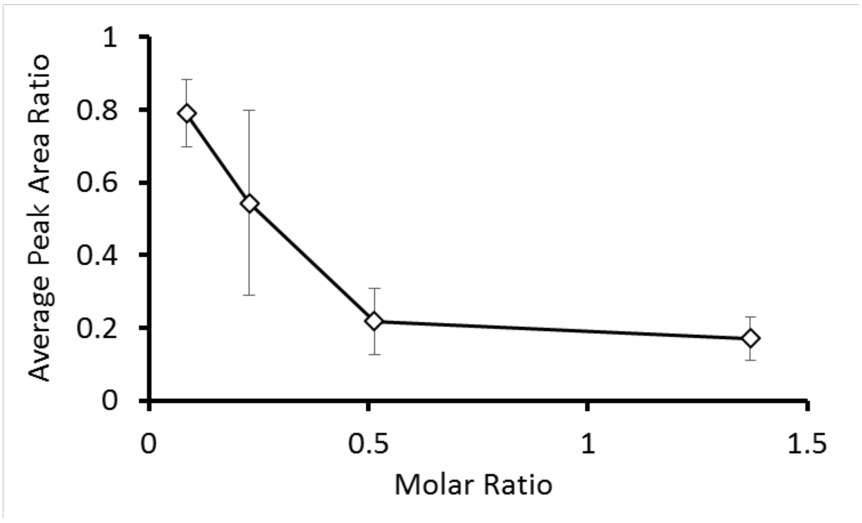

SI Figure 2 Plotted average peak area ratios against the molar ratio of HAP: Brushite, n = 10

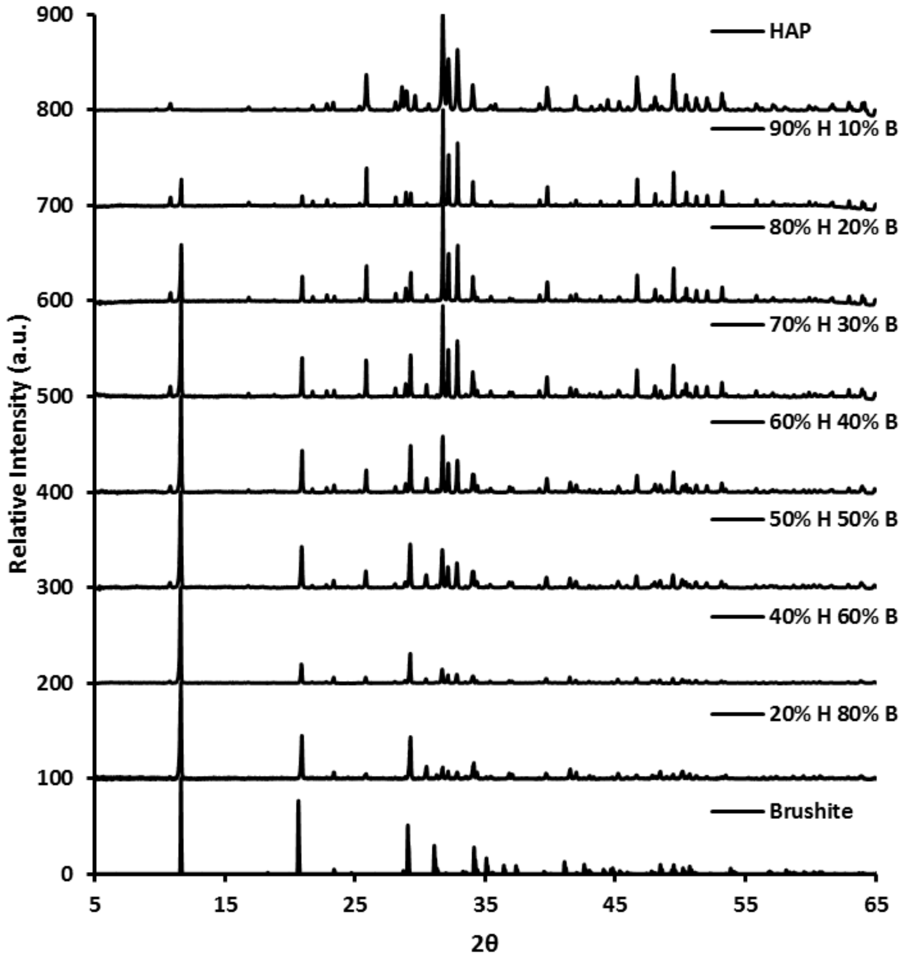

SI Figure 3 Example XRD patterns for the HAP and brushite %/wt mixtures.

A

	Sequence	Net charge at pH 7.4	Average Hydrophobicity	pI
CaP(H)	SVSVGMKPSPRP	+2	+13.87	11.71
CaP(S)	STLPIPHEFSRE	-1	+16.67	5.23
CaP(V)	VTKHLNQISQSY	+1	+13.05	9.54

B

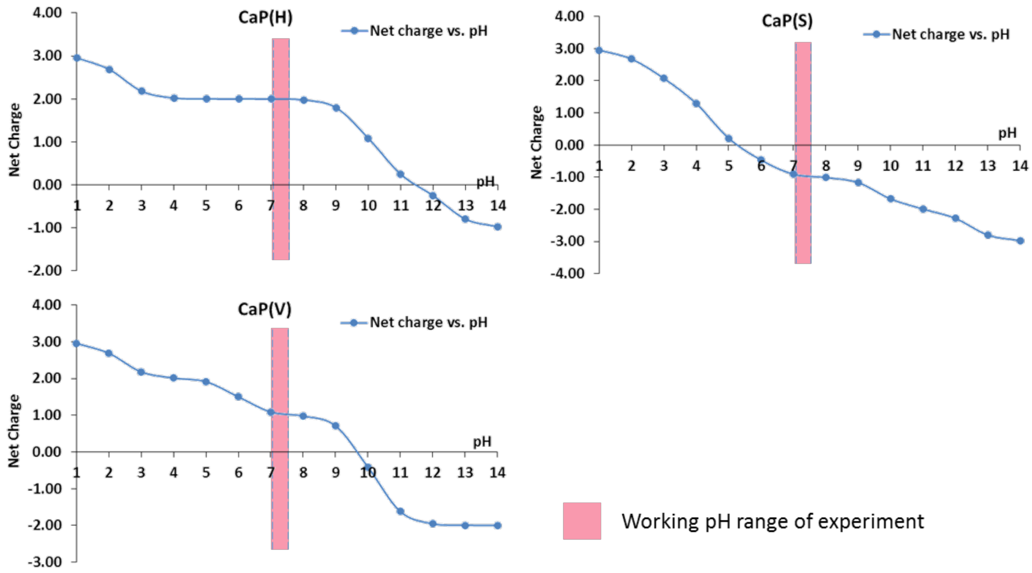

C

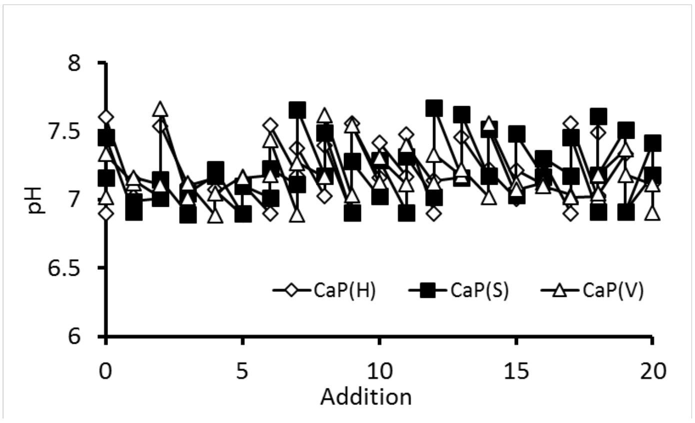

SI Figure 4 Calculated peptide properties for the three HAP binding peptides included in the study. A) Tabulated physical property data for each peptide B) Graphical representations of peptide net charges with response to pH. C) pH of the peptide mineralising solution after each addition of 200 µL 120 mM sodium phosphate dibasic and 200 µL 200 mM calcium chloride.

%/wt HAP			
% HAP	Actual	ICP	XRD
0	0.00	0.00	0.00
20	20.27	14.83	20.73
40	40.62	19.98	46.36
60	61.87	64.80	60.18
80	80.21	77.73	79.96
100	100.00	99.38	100.00

SI Table 1 *All techniques used to analyse HAP/Brushite standards, displayed as a percentage of HAP by weight. Data is stated as an average, ICP: N=3 and XRD: N=10*
